# An Integrative Analysis to Identify Driver Genes in Esophageal Squamous Cell Carcinoma

**DOI:** 10.1371/journal.pone.0139808

**Published:** 2015-10-14

**Authors:** Genta Sawada, Atsushi Niida, Hidenari Hirata, Hisateru Komatsu, Ryutaro Uchi, Teppei Shimamura, Yusuke Takahashi, Junji Kurashige, Tae Matsumura, Hiroki Ueo, Yuki Takano, Masami Ueda, Shotaro Sakimura, Yoshiaki Shinden, Hidetoshi Eguchi, Tomoya Sudo, Keishi Sugimachi, Makoto Yamasaki, Fumiaki Tanaka, Yuji Tachimori, Yoshiaki Kajiyama, Shoji Natsugoe, Hiromasa Fujita, Yoichi Tanaka, George Calin, Satoru Miyano, Yuichiro Doki, Masaki Mori, Koshi Mimori

**Affiliations:** 1 Department of Surgery, Beppu Hospital, Kyushu University, 4546, Tsurumihara, Beppu 874-0838, Japan; 2 Department of Gastroenterological Surgery, Graduate School of Medicine, Osaka University, 2-2 Yamadaoka, Suita 565-0871, Japan; 3 Laboratory of DNA Information Analysis, Human Genome Center, Institute of Medical Science, University of Tokyo, 4-6-1 Shirokanedai, Minato-ku, Tokyo 108-8639, Japan; 4 Department of Surgery, National Cancer Center Hospital, Tokyo, Japan; 5 Department of Esophageal and Gastroenterological Surgery, Juntendo University School of Medicine, Tokyo, Japan; 6 Department of Surgical Oncology and Digestive Surgery, Kagoshima University School of Medicine, Kagoshima, Japan; 7 Department of Surgery, Kurume University School of Medicine, Kurume, Japan; 8 Division of Gastroenterological Surgery, Saitama Cancer Center, Saitama, Japan; 9 Department of Experimental Therapeutics and The Center for RNA Interference and Non-Coding RNAs, The University of Texas MD Anderson Cancer Center, 1515 Holcombe Boulevard, Houston, Texas, United States of America; Peter MacCallum Cancer Centre, AUSTRALIA

## Abstract

**Background:**

Few driver genes have been well established in esophageal squamous cell carcinoma (ESCC). Identification of the genomic aberrations that contribute to changes in gene expression profiles can be used to predict driver genes.

**Methods:**

We searched for driver genes in ESCC by integrative analysis of gene expression microarray profiles and copy number data. To narrow down candidate genes, we performed survival analysis on expression data and tested the genetic vulnerability of each genes using public RNAi screening data. We confirmed the results by performing RNAi experiments and evaluating the clinical relevance of candidate genes in an independent ESCC cohort.

**Results:**

We found 10 significantly recurrent copy number alterations accompanying gene expression changes, including loci 11q13.2, 7p11.2, 3q26.33, and 17q12, which harbored *CCND1*, *EGFR*, SOX2, and *ERBB2*, respectively. Analysis of survival data and RNAi screening data suggested that *GRB7*, located on 17q12, was a driver gene in ESCC. In ESCC cell lines harboring 17q12 amplification, knockdown of *GRB7* reduced the proliferation, migration, and invasion capacities of cells. Moreover, siRNA targeting *GRB7* had a synergistic inhibitory effect when combined with trastuzumab, an anti-*ERBB2* antibody. Survival analysis of the independent cohort also showed that high *GRB7* expression was associated with poor prognosis in ESCC.

**Conclusion:**

Our integrative analysis provided important insights into ESCC pathogenesis. We identified *GRB7* as a novel ESCC driver gene and potential new therapeutic target.

## Introduction

Esophageal squamous cell carcinoma (ESCC) is a relatively common type of malignant cancer in East Asian countries, including Japan [1], and is highly aggressive due to the frequent involvement of lymph node metastasis and tumor invasion to adjacent organs at early stages [2]. Recently, advancements in therapeutic modalities have improved clinical outcomes to some extent, although the 5-year survival rate of ESCC patients still remains at only 30%–40% [3–6].

Copy number aberrations (CNAs) and accompanying dysregulation of gene expression are known to play a critical role in the pathogenesis of human cancers [7]. Aberrant genomic regions can be used for clue to find oncogenes or tumor suppressor genes. Furthermore, integration of DNA copy number data and gene expression data could more efficiently identify driver genes. Such integrative analyses have been performed on a number of cancers [8–10], and there are a few on ESCC [11, 12].

Here, we screened for ESCC driver genes by combining gene copy number and expression data. We also refined the candidate list by performing survival analysis on the expression data and testing genetic vulnerability using public RNAi screening data. This series of analyses suggest that *GRB7*, located on 17q12, was overexpressed due to copy number gains and play a critical role in tumor growth and invasion. Although tumor-promoting role of *GRB7* in ESCC has been previously suggested in a few reports [13–15], in the present study, the significance of *GRB7* in ESCC was firmly confirmed by the integrative analysis of gene expression and copy number. Furthermore, we verified biological functionality of *GRB7* by siRNA-mediated knockdown experiments, and also validated that high *GRB7* expression was associated with poor survival in an independent ESCC cohort. Collectively, this study suggests that *GRB7* may be a novel therapeutic target for the treatment of ESCC.

## Material and Methods

The protocol of this study protocol was reviewed and approved by Kyushu University (Fukuoka, Japan), Juntendo University (Tokyo, Japan), National Cancer Center Hospital (Tokyo, Japan), Kurume University (Kurume, Japan), Saitama Cancer Center (Saitama, Japan), and Kagoshima University (Kagoshima, Japan). Approval number from Institutional Review Board (IRB) is 395–02.

### Clinical samples

Between January 2000 and December 2008, 168 tissue samples from patients with ESCC were collected from six hospitals (Juntendo University Hospital, National Cancer Center Hospital, Kurume University Hospital, Saitama Cancer Center, Kagoshima University Hospital, and Kyushu University Hospital). All participants provided written informed consent and all procedures were approved by IRB of each institution.

The 168 samples were divided into 2 groups: the discovery set, which included 83 patients, 78 of whom were assigned for microarray analysis and 62 of whom were included in aCGH analysis; and the validation set, which included the remaining 85 patients. Experimental information of 83 patients from the discovery set is shown in S1 Fig, S1 and S2 Tables. Information on the validation set is shown in S3 Table. The survival analysis of clinical samples was performed based on gene expression rather than copy number because RNAi screening data was used to narrow down ESCC candidate driver genes and the functionality of *GRB7* was also estimated by siRNA-mediated knockdown experiments.

### Cell culture

TE4 and KYSE410 cells were provided by the American Type Culture Collection. These cell lines were authenticated by short tandem repeat profiling using the GenePrint 10 System (Promega, WI, USA). Cells were maintained in RPMI-1640 containing 10% fetal bovine serum (FBS) with 100 U/mL penicillin and 100 mg/mL streptomycin and cultured in a humidified 5% CO_2_ incubator at 37°C.

### Laser microdissection (LMD)

Tissue specimens from the discovery set were embedded in Tissue-Tek OCT compound (Sakura Fineteck USA, Torrance, CA, USA) and sectioned using an LMD system (Leica Laser Microdissection System, Leica Microsystems, Wetzlar, Germany) as previously described [16]. For LMD, 8-μm frozen sections were fixed in 70% ethanol for 30 s, stained with hematoxylin and eosin, and dehydrated for 5 s each in 70%, 95%, and 100% ethanol with a final 5 min in xylene. Sections were air-dried and then microdissected with the LMD system. Target cells were excised, with each section having at least 100 cells, and bound to transfer film. Total DNA and RNA were then extracted.

### Array-CGH and Copy number analysis

Genomic DNA of sixty-two microdissected tumor samples and three normal samples was isolated using a QIAamp DNA Micro Kit (Qiagen, Valencia, CA, USA). After Labeling and hybridization of genomic DNA onto the Agilent Human Genome Microarray Kit 244K (Agilent Technologies), the log ratios to the reference DNA (hg19) were obtained and segmented using the Circular Binary Segmentation algorithm [17, 18]. To identify recurrent CNAs, we applied the GISTIC algorithm [19, 20] to the aCGH data with default parameter settings for amplitude threshold, cutoff q-value, and confidence interval. No filtering for copy number polymorphisms (CNPs) was done and thus there existed the possibility that their minimal regions were CNPs. Using the University of California Santa Cruz genome browser (https://genome.ucsc.edu/), we confirmed that significant regions less than 500kb was not due to CNPs. The data was registered in Gene Expression Omnibus (GEO; accession number: GSE47630). In addition, a public copy number dataset (GEO accession number: GSE17958) was utilized to validate our data [21].

### Expression array

Total RNA from 78 microdissected tumor samples and three samples from normal esophageal mucosa were extracted using ISOGEN (Nippon Gene), and cDNA was synthesized from 8.0 μg of total RNA. Cyanine (Cy)-labeled cRNA was prepared using T7 linear amplification, fragmented and hybridized to an oligonucleotide microarray (Whole Human Genome 4x44 Agilent G4112F). Fluorescence intensities were obtained using an Agilent DNA microarray scanner, and subject to quantile normalization. The data was registered as GSE47404.

### Integrative analysis

**Fig 1:**
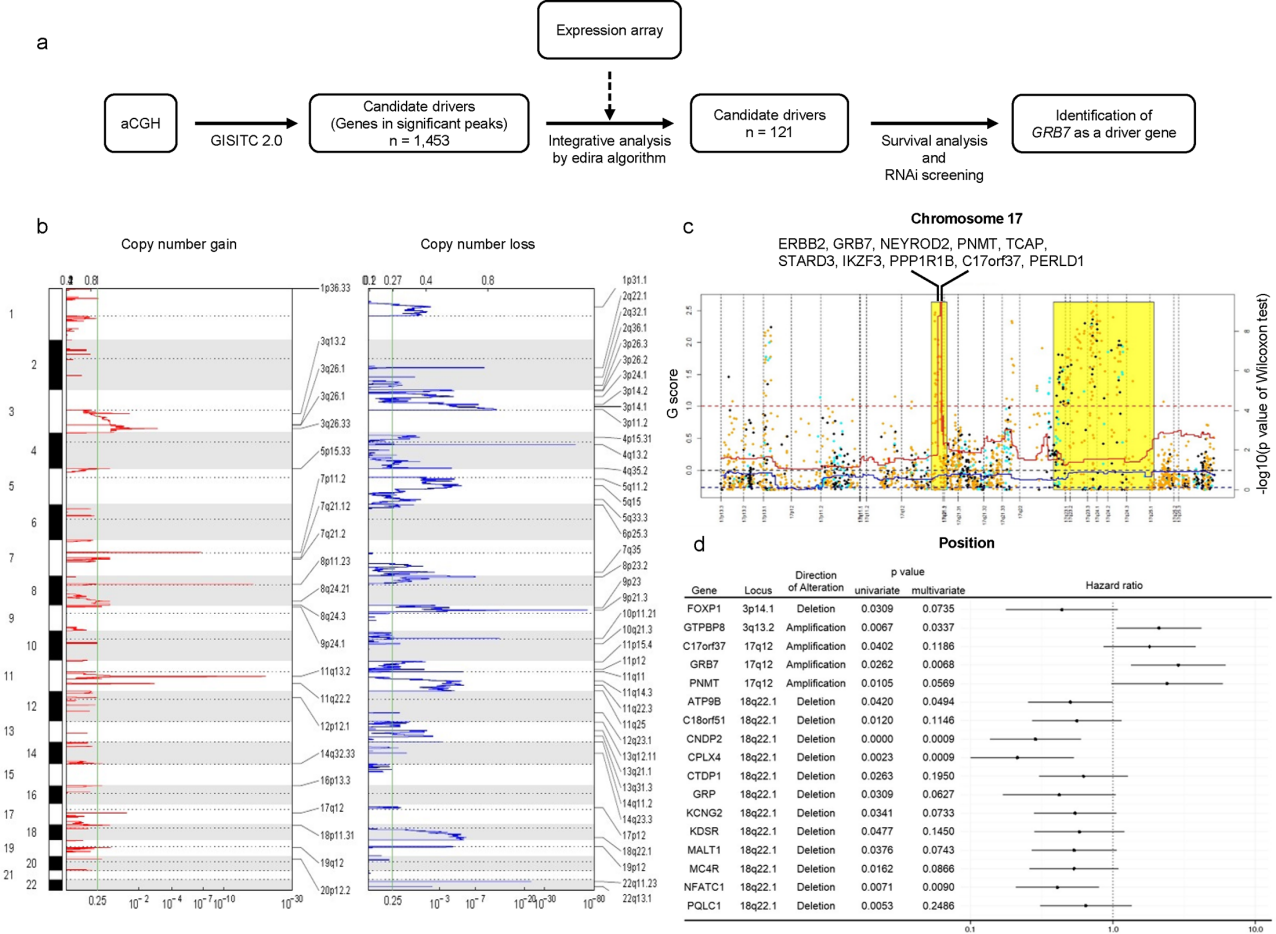
Integrative analysis and candidate genes. (a) Schema of the analysis flow in this study. (b) Plots of recurring high-level amplifications and deletions in 62 ESCCs from GISTIC analysis. The X-axis shows the G score (top) and false discovery rate (q value; bottom) for recurrent peaks across the genome, with the green line indicating an arbitrary false discovery rate (FDR) cutoff of 0.25. Labels on the right denote the positions of peaks in significantly altered regions. (c) Results of integrative analysis and G scores obtained from GISTIC analysis in chromosome 17. The y-axis on left side represents the G score, while the y-axis on the right side represents the p-value from Wilcoxon tests using the edira algorithm. The G score of the positive value indicates amplification, while that of the negative value indicates deletion. The red and blue lines show G scores of amplifications and deletions, respectively. The dotted red line indicates the threshold of amplification, and the dotted blue line indicates the threshold of deletion. Dot plots indicate p-values from Wilcoxon tests in each position. Dot plots in light blue represent deletions, and dot plots in orange represent amplifications. The regions in yellow indicate significant correlations between expression and copy number alterations. (d) Forest plot of hazard ratios of candidate genes for survival. The table on the left side of the Forest plot shows the p-value from univariate and multivariate survival analysis for each gene.

Our analysis flow in this study is summarized in Fig 1a. To identify differentially expressed genes associated with CNAs in ESCC, we applied gene expression and aCGH data to the edira algorithm [22]. This algorithm is characterized as the bivariate approach with the assessment of the equally directed abnormality of copy number and gene expression. Instead of a two-step approach in which initially regions with CNAs are detected and then the expression levels of genes in these regions are separately investigated, copy number and gene expression are estimated simultaneously in the bivariate approach. Furthermore, the equally directed abnormalities of both copy number and expression (concordant changes of both variables) are assessed in comparison with reference data. In the present study, gains and losses were defined as log2 ratio of 0.1 or more and of −0.1 or less, respectively. For detection of DNA regions displaying equally directed abnormalities, p-values for the Wilcoxon test were set as 10–6.

### Analysis of RNA interference screening data

RNAi screening data produced in Project Achilles by the Broad institute was downloaded from http://www.broadinstitute.org/achilles [23]. In the data set, growth inhibitory effects of 54,020 shRNAs targeting 11,194 genes were measured in 102 human cancer cell lines. Since each gene is targeted by multiple shRNAs, we summarized the effects of the shRNA on each gene using a Kolmogorov-Smirnov statistic; for each cell line, shRNAs were ranked according to their inhibitory effects on proliferation, and enrichment of shRNAs targeting a gene of interest in higher ranking is scored as done for gene set analyses [24]. A p-value for the Kolmogorov-Smirnov statistic was calculated by a permutation tests, and we reported the minus-log-scaled p-value as a vulnerability score. We searched for genes that show genetic vulnerability specifically in ESCC based on the vulnerability scores; for each gene, a t-test was performed to test the difference of vulnerability scores between 9 ESCC cell lines and others. We assumed genes with q-values above 0.1 as significant.

### Real-time quantitative reverse transcription (RT)-PCR

The primer sequences for real-time RT-PCR were as follows: *GRB7*, forward, 5′-TGCAAAGTTTGTGAGTGGTGA-3′ and reverse, 5′-GAACGTAGGCAGTATGGTTTCC-3′; *ERBB2*, forward, 5′-CCCAGGGAGTATGTGAATGC-3′ and reverse, 5′-CAGGCCACACACTGGTCA-3′; and *GAPDH* forward, 5′-GTCAACGGATTTGGTCTGTATT-3′ and reverse, 5′-AGTCTTCTGGGTGGCAGTGAT-3′. The real-time quantitative monitoring of PCR assays was performed using LightCycler System (Roche Applied Science, Indianapolis, IN, USA) and LightCycler 480 Probes Master kit (Roche Applied Science). *GRB7* and *ERBB2* expression levels were normalized to *GAPDH* expression levels.

### RNA interference experiments

Stealth RNA siRNA targeting human *GRB7* and *ERBB2* and negative control siRNA were purchased from Invitrogen (Carlsbad, CA, USA). KYSE410 and TE4 cells were transfected with siRNA at a concentration of 20 μmol/L using Lipofectamine RNAiMAX (Invitrogen) and incubated in glucose-free Opti-MEM (Invitrogen). Total RNA from transfected cell lines was extracted using a QIAamp DNA Micro Kit (Qiagen).

### Protein expression analysis

From cells grown to semiconfluence, total protein was extracted in radioimmunoprecipitation assay buffer (Thermo Fisher Scientific, Inc. Rockford, IL, USA) 48 hours after transfection. Aliquots of total protein (12 mg) were electrophoresed on 10% sodium dodecyl sulfate (SDS) polyacrylamide gels (Tris-HCl gels; Bio-Rad Laboratories Inc., Hercules, CA, USA). Following electrophoresis, the separated proteins were transferred to polyvinylidene difluoride membranes (Millipore Co., Billerica, MA, USA). ERBB2 and GRB7 proteins were detected using an anti-ERBB2 rabbit monoclonal antibody (Epitomics, Inc) at a 1:5000 dilution and an anti-GRB7 rabbit monoclonal antibody (Gene Tex International Corporation) at a 1:1000 dilution. The levels of each protein were normalized to the level of β-actin protein, which was detected using anti-β-actin antibodies at a 1:200 dilution.

### Proliferation assay

KYSE410 and TE4 cells were transfected with siRNA targeting *GRB7* or *ERBB2* or with negative control siRNA. Cells were then seeded at 8.0 × 10^3^ cells per well in 96-well flat-bottomed microtiter plates in a final volume of 100 μL of culture medium per well. Cells were incubated in a humidified atmosphere (37°C and 5% CO_2_) for 48 and 96 h after transfection. The 3-(4, 5-dimethylthiazol-2-yl)-2, 5-diphenyltetrazolium bromide (MTT) assay (Roche Diagnostics Corp.) was used to measure cell growth inhibition. After incubation, 10 μL of MTT labeling reagent (final concentration of 0.5 mg/mL) was added to each well, and the plate was incubated for 4 h in a humidified atmosphere. Solubilization solution (100 μL) was added to each well, and the plate was incubated overnight in a humidified atmosphere. After confirming that the purple formazan crystals were completely solubilized, the absorbance of each well was measured by a Model 550 series microplate reader (Bio-Rad Laboratories), at a wavelength of 570 nm corrected to 655 nm. The assay was performed using 6 replicates.

### Migration assay

The BD Falcon FluoroBlok 24 Multiwell Insert System (BD Bioscience, San Jose, CA) was used to evaluate invasive capacity. Twenty-four hours before the assay, cells were transfected with *GRB7*- and *ERBB2*-specific siRNAs or a negative control siRNA. The cells (1.0 × 10^5^ cells/500 μL/well) were then placed in the upper chamber of the 24-well plate with serum-free medium. The lower chamber was filled with 750 μL of medium containing 10% FBS, which acted as a chemo-attractant, and plates were incubated in a humidified atmosphere (37°C and 5% CO_2_). After a 72-h incubation, the upper chamber was transferred into a second 24-well plate containing 500 μL/well calcein AM (4 μg/mL) in Hank’s balanced salt solution (HBSS) and incubated for an additional 1 h (37°C and 5% CO_2_). Invasive cells that migrated through the membrane were evaluated using a fluorescence plate reader with excitation/emission wavelengths of 485/535 nm. Migration was measured as the percentage of fluorescence emission compared to that of an invasive fibrosarcoma cell line (HT-1080), which served as a control. Each independent experiment was performed 3 times.

### Invasion assay

The BD BioCoat Tumor Invasion System, 24 Multiwell (BD Bioscience) was used to evaluate invasive capacity. Twenty-four hours before the assay, cells were transfected with *GRB7*- and *ERBB2*-specific siRNAs or a negative control siRNA. The upper chamber of the 24-well plate was pre-incubated with 500 μL of phosphate-buffered saline (PBS) for 2 h at 37°C. After removing the PBS, the cells (1.0 × 10^4^ cells/500 μL/well) were placed in the upper chamber of the 24-well plate with serum-free medium. The subsequent analysis was performed according to the methods described for the migration assay above.

### Statistical analysis

The significance of differences between 2 groups was estimated with the Student’s t-test, chi-squared test and Fisher’s exact test. Overall survival curves were plotted according to the Kaplan-Meier method, and their p-values were calculated by the log-rank test. Variables with a p-value of less than 0.05 by univariate analysis were used in subsequent multivariate analysis based on the Cox proportional hazards model. All differences were considered statistically significant at the level of P < 0.05. Statistical analyses were conducted in the R platform.

## Results

### CNAs in ESCC

Our analysis of aCGH data showed that the most significant amplification peak in ESCC was located on chromosome segment 11q13.2, which harbored *CCND1* (Fig 1b). Additional amplification peaks with high significance (GISTIC q-values < 0.05) were found on 8p11.23, 7p11.2, 3q26.33, and 17q12 (Fig 1b). Those segments contain well-known oncogenes, including *EGFR*, *SOX2*, and *ERBB2*, which are registered in the Cancer Gene Census database as amplified genes in several types of cancer [25]. The most significant deletion peak was observed at chromosome segment 9p21.3, containing *CDKN2A* (Fig 1b). We also identified significant arm-level events, which included arm-level deletion of 3p and 9p, and amplification of 20q (S4 Table).

In addition to our aCGH data, a public copy number data set registered in GSE17958 was used to detect recurrent significant peaks of copy number changes. ESCC clinical sample data, comprising a total of 30 samples, were extracted from these data and analyzed in the same manner. Subsequently, we confirmed recurrent significant amplification peaks of *SOX2* on 3q26.33, *EGFR* on 7p11.2, *MYC* on 8q24.21, *CCND1* on 11q13.2, and *ERBB2* on 17q12 (S2 Fig). Moreover, deletion peaks of *CDKN2A* on 9p21.3 and *FHIT* on 3p14.2 were also observed in the GEO data set.

### Integrative analysis of copy number and gene expression data

To determine the biological relevance of CNAs detected by GISTIC analysis, we performed integrative analysis of copy number and gene expression data from 57 ESCC samples using the edira algorithm (S1 Fig) [22]. We summarized the results of G scores obtained from GISTIC analysis and results of integrative analysis in the same plot. Fig 1c shows the result about chromosome 17, where the region with the amplification peak of the associated G score was matched with the region identified by the edira algorithm as abnormal toward equal directions, marked by a yellow background in the plot. This result indicated that genes on segment 17q12, such as *ERBB2* and *GRB7*, were overexpressed by genomic amplification. We also found that upregulation of *CCND1*, *EGFR*, and *SOX2* and downregulation of *SMAD4*, *FHIT*, and *TGFBR2* resulted from CNAs (Table 1 and S3 Fig). Collectively, these results raised a possibility that copy number aberrations of these genes are driver events in ESCC development.

**Table 1:** GISTIC-defined peaks associated with altered expression in ESCC.

Cytoband	Q value	Wide peak boundaries	Direction of aberration	Frequency of aberration (%)	Significance in GSE17958	Genes in peak	Candidate targets		
11q13.2	1.58E-20	chr11:69034285–69149286	amplification	80.6	significant	1	CCND1		
8p11.23	6.02E-17	chr8:39356625–39535683	amplification	90.3		1			
7p11.2	1.65E-07	chr7:54816792–55162089	amplification	61.3	significant	1	EGFR		
3q26.33	0.00098284	chr3:182878001–183294550	amplification	85.5	significant	1	SOX2		
3q13.2	0.023964	chr3:113499996–114212594	amplification	67.7		9	CD200	ATG3	
17q12	0.031192	chr17:35017165–35202671	amplification	58.0	significant	10	ERBB2	GRB7	
18q22.1	1.90E-06	chr18:45971551–76117153	deletion	71.0		93	BCL2	SMAD4	NFATC1
3p14.2	0.00073274	chr3:59007938–61527556	deletion	93.5	significant	1	FHIT		
3p24.1	0.0031654	chr3:30021139–31677792	deletion	91.9		3	TGFBR2		
3p14.1	0.040761	chr3:70098576–71732543	deletion	91.9		1	FOXP1		

Q value indicated the significance of copy number alteration in each region.

The % aberration included any gain over 0.1 or loss under −0.1 (log2 ratio).

Candidate targets were genes that have actual correlations between expression and copy number alterations in 'genes in peak'.

### Analysis of the associations between candidate genes and survival

To evaluate the clinical significance of candidate genes detected by integrative analysis, we performed survival analysis of the discovery set on expression of all 121 genes which were identified as 'genes in peak' (Table 1). In univariate analysis, we found 17 significant genes, located on 3p14.1, 3q13.2, 17q12, and 18q22.1 (Fig 1d); these genes included cancer-related genes, such as *FOXP1*, *GRB7*, and *NFATC1*, indicating that altered expression of specific genes through CNAs could influence the clinical course of patients. *ERBB2*, a gene reported to be associated with prognosis in several cancers, did not show any significance in our ESCC cases. Multivariate analysis also showed that 6 genes, including *GRB7*, were independent prognostic factors for survival in patients with ESCC (Fig 1d).

### Analysis of RNA interference screening data

Knockdown experiments are needed to prove functionality of the amplified and overexpressed genes. For this purpose, we utilized a public RNAi screening dataset from Project Achilles by the Broad institute that measures knockdown effects of 11,194 genes on proliferation of 102 human cancer cell lines. We searched for genes that show genetic vulnerability lineage-specifically in ESCC cell lines. It was reported that a similar approach successfully identified a lineage-specific driver gene in ovarian cancer [23]. We found that three genes (*ERG*, *GRB7*, and *HLF*) were lineage-specifically essential for the growth of 9 ESCC cell lines (Table 2). Taken together with above-stated data and the result of the analysis of RNAi screening data, *GRB7* was the only candidate gene which was recurrently identified. Therefore, these results prompted us to experimentally test the biological importance of *GRB7*.

**Table 2:** Results of analysis on RNA interference screening data of Project Achilles.

Gene		Locus	P value	Q value
*ERG*	v-ets erythroblastosis virus E26 oncogene homolog	21q22.2	3.44E-07	0.0054
*GRB7*	Growth factor receptor-bound protein 7	17q12	1.69E-05	0.084
*HLF*	Hepatic leukemia factor	17q22	2.10E-05	0.0927

### Validation of the significance of *GRB7* in ESCC by siRNA-mediated knockdown

**Fig 2:**
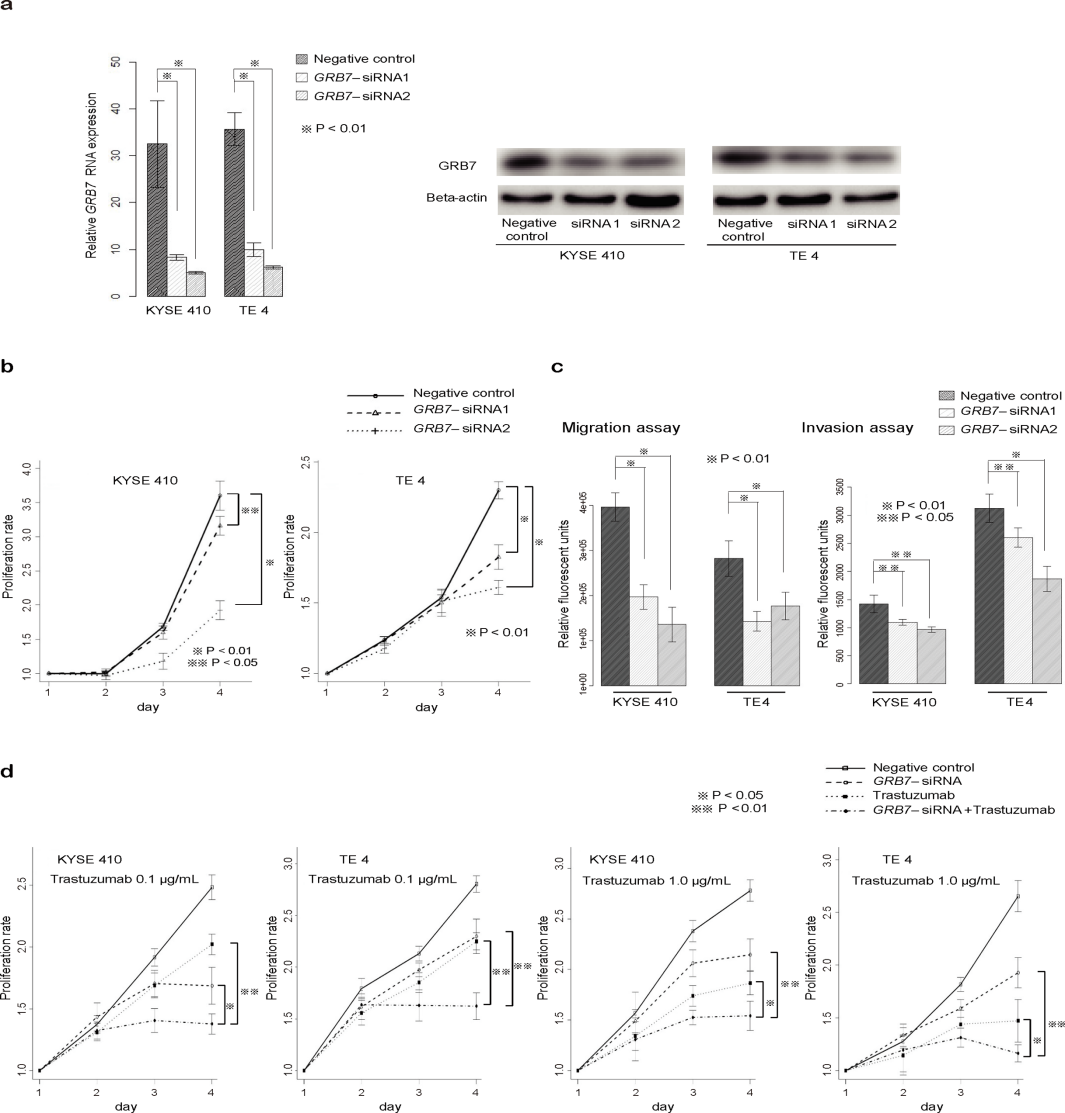
Knockdown of *GRB7* expression in ESCC cell lines. (a) Reductions in mRNA and protein levels of GRB7 at 48 hours after siRNA transfection in KYSE410 and TE4 cells. The results are the mean ± SD from 3 replicates of a single experiment. (b) *GRB7* inactivation reduced proliferation of KYSE410 and TE4 cells. Cell growth was measured on days 2, 3, and 4 by MTT assay. Absorbance at day 0 was assigned a value of 1. The results are the mean ± SD from 6 replicates of a single experiment. (c) Migration and invasion assays using *GRB7*-knockdown cells. Each bar represents the average of 3 measurements. (d) Inhibitory effects of siRNA targeting *GRB7* in combination with trastuzumab. Cells were transfected with siRNA targeting *GRB7* or negative control siRNA and treated with or without trastuzumab (0.1 and 1.0 μg/mL). Cells were then seeded in 96-well plates, and cell growth was monitored every 24 hours using MTT assays. Absorbance at day 0 was assigned a value of 1. The results are the mean ± SD from 6 replicates of a single experiment.

To validate the role of *GRB7* in the growth of ESCC cell lines, *GRB7* expression was suppressed by transient siRNA transfection in KYSE410 and TE4 cells, chosen because both harbor amplification of 17q12 and show high *GRB7* mRNA expression among ESCC cell lines (data not shown). The reduction of *GRB7* expression was confirmed by quantitative real-time RT-PCR and western blotting (Fig 2a). Consistent with the results from analysis of RNAi screening data, siRNA-mediated knockdown of *GRB7* reduced the proliferation of KYSE410 and TE4 cells (Fig 2b). Furthermore, siRNA-mediated knockdown of *GRB7* suppressed the invasive and migratory abilities of ESCC cells as compared to cells transfected with a negative control siRNA (Fig 2c).

A well-established oncogene *ERBB2* also resides in 17q12; therefore, we similarly confirmed the biological significance of *ERBB2* in ESCC cell lines. The inhibition of *ERBB2* expression by siRNA transfection was performed (Figure A in S1 File), and knockdown of *ERBB2* expression reduced the growth rate of both KYSE410 and TE4 cells (Figure B in S1 File). Moreover, we found that trastuzumab, an anti-HER2 antibody, had an inhibitory effect on the growth of the ESCC cell lines (Figure C in S1 File)

Next, we investigated the synergistic effects between *ERBB2* and *GRB7* on cell proliferation. The combination of trastuzumab (0.1 μg/mL) plus transfection of siRNA targeting *GRB7* had a stronger inhibitory effect on cell growth than administration of trastuzumab or siRNA targeting *GRB7* alone (Fig 2d). This was observed under a high concentration of trastuzumab (1.0 μg/mL), which indicated that knockdown of *GRB7* expression had a synergistic inhibitory effect on ESCC cell lines with amplification of 17q12.

### Validation of the clinicopathological significance of *GRB7* mRNA expression in ESCC

**Fig 3:**
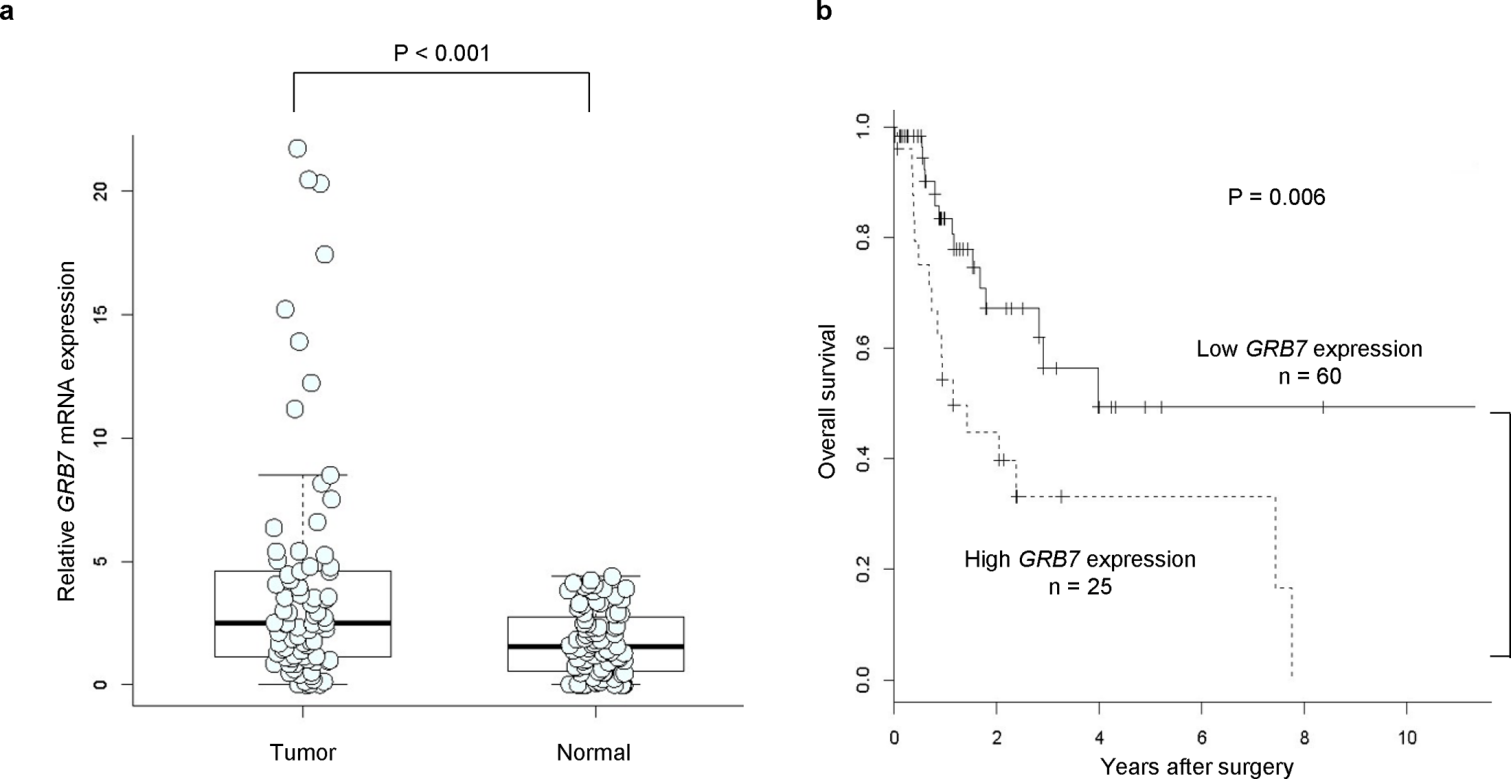
Clinical significance of *GRB7* mRNA expression in ESCC is validated in the validation set. (a) Analysis of *GRB7* mRNA expression in tumor tissues and the corresponding normal mucosa by real-time RT-PCR. (b) Kaplan-Meier survival curves for ESCC patients according to *GRB7* mRNA expression.

Finally, we evaluated *GRB7* mRNA expression in the validation set. First, to validate *GRB7* overexpression in ESCC tumor tissue, we compared *GRB7* mRNA expression levels in tumor tissues with corresponding normal mucosa. Consistent with the results of integrative analysis, *GRB7* was significantly upregulated in tumor tissues compared to the corresponding normal mucosa (Fig 3a). We then analyzed the association between clinicopathological factors and *GRB7* expression. As shown in the discovery set (S2 Table), clinicopathological factors in the high *GRB7* expression group were not significantly different from those in the low *GRB7* expression group (S5 Table). With regard to overall survival, patients with high *GRB7* expression had a significantly poorer prognosis than those with low *GRB7* expression (P = 0.006; Fig 3b). Univariate analysis revealed that the level of *GRB7* expression, histology (well or moderate/poor), and the presence of lymph node metastasis were significantly correlated with prognosis (Table 3). These factors identified by univariate analysis were then applied to multivariate analysis, and *GRB7* expression level showed marginal significance (P = 0.08) for the prognosis of ESCC patients in multivariate analysis. These results were consistent with those of the discovery set.

**Table 3:** Results of univariate and multivariate analysis of clinicopathlogical factors for 5-year overall survival in the validation set.

Factors (number of patients)	Univariate analysis				Multivariate analysis			
	Hazard ratio	95% CI low	95% CI high	P value	Hazard ratio	95% CI low	95% CI high	P value
Age (70< / ≥70: n = 59 / 26)	1.17	0.59	2.33	0.65	-	-	-	-
Depth (T1 / T2-T4: n = 9 / 76)	3.07	0.73	12.96	0.13	-	-	-	-
Venous invasion (Negative / Positive: n = 10 / 75)	-	0.00	-	1.00	-	-	-	-
Histology (well / mod&poor: n = 30 / 55)	2.32	1.04	5.19	0.04	1.43	0.58	3.52	0.44
Lymph node metastasis (Negative / Positive: n = 23 / 62)	3.55	1.34	9.40	0.01	2.23	0.71	6.96	0.17
*GRB7* mRNA expression (Low / High: n = 60 / 25)	2.54	1.28	5.05	0.01	1.91	0.92	3.96	0.08

CI: confidence interval, well: well differentiated squamous cell carcinoma, mod: moderately differentiated squamous cell carcinoma, poor: poorly differentiated squamous cell carcinoma.

## Discussion

The integrative analysis of copy number and gene expression data identified a number of significant genes that may be associated with the pathogenesis of ESCC. Moreover, by mining RNAi screening and survival data, we identified *GRB7* as a candidate oncogene. Although linage specific analysis in RNAi data excluded genes that are important drivers for multiple cancers as well as ESCC, such as *ERBB2* and *EGFR*, through knockdown assays in ESCC cell lines and additional survival analysis, we confirmed *GRB7* as a new driver gene in ESCC.

Several studies have described genomic alterations occurring in ESCC. Similar to other cancers, recurrent amplification of 7p11.2, 8q24.21, and 11q13.2, harboring *EGFR*, *MYC*, and *CCND1*, has previously been reported in ESCC [12, 21, 26, 27]. Genomic amplification of 3q26.33, which harbors the gene encoding the transcription factor *SOX2*, has been also found in ESCC. Compared with esophageal adenocarcinoma, the estimated frequency of *SOX2* amplification was reported to be significantly higher in ESCC [28]. The loss of heterozygosity (LOH) of 3p loci, including *FHIT*, *VHL*, and *RASSF1A* (known tumor suppressors), has been confirmed in ESCC [29], and loss of *FHIT* has been reported to be associated with poor prognoses [30]. Our findings in this study are concordant with the results of those previous studies. Additionally, we found additional genes previously not reported in any genome-wide studies in ESCC, such as *FOXP1* and *NFATC1*, which were downregulated due to deletion. Low *FOXP1* expression has been reported to be associated with poor prognosis in non-small cell lung cancer [31], while *NFAT1* has been reported to act as a tumor suppressor gene [32]. Our analyses provided significant insights into the molecular biology of ESCC.

The significance of HER2 amplification at 17q12 in ESCC has been highlighted in previous studies. The frequency of amplification of HER2 in ESCC ranges from 3.9% to 41.4% [28, 33–36]. Several studies have suggested that HER2 expression is associated with various clinicopathological factors and poor prognosis. Furthermore, investigation of the therapeutic efficacy of trastuzumab in patient-derived esophageal squamous cell carcinoma xenografts has been performed [37].

On the other hand, although several studies have reported the oncogenic role of *GRB7* expression in ESCC cells [13–15], few studies have focused on CNAs of *GRB7*. *GRB7* is an adaptor protein that relays signals from cell surface receptors to specific downstream signaling cascades via protein-protein interactions of a variety of tyrosine kinases with its Src-homology 2 (SH2) domain [38–40]. Several studies have demonstrated that *GRB7* is connected to cell motility through its association with focal adhesion kinase, phosphoinositides, ephrin receptor, and calmodulin [41–43]. Moreover, a previous study suggested that *GRB7* affects both the proliferative and invasive potential of HER2+ breast cancer cells through direct binding with HER2 and FAK [44]. Additionally, *GRB7* promotes tumorigenesis through the formation of a novel EGFR-GRB7-Ras signaling complex [45]. Therefore, given its important roles as a signal transduction molecule in the activation of oncogenic signaling pathways, numerous studies have attempted to develop inhibitors targeting the SH2 domain of GRB7 in order to inhibit the aberrant activation of related signaling activities and eliminating cancer cells [46–49].

In this study, we also confirmed that trastuzumab had an inhibitory effect on ESCC cell lines with 17q12 amplification. Therefore, in addition to its applications in breast and gastric cancers, trastuzumab may be an effective drug for the treatment of patients with ESCC harboring amplification of 17q12. Moreover, when combined with trastuzumab, knockdown of *GRB7* had a synergistic inhibitory effect on cell proliferation. These data suggest that *GRB7* and *ERBB2*, two genes residing in close loci, are co-amplified and synergistically functions as driver genes in ESCC. This result reminds us of recent studies in lung squamous cell carcinoma [50] and breast cancer [51], which identified that two co-amplified genes in close loci function as synergistic driver genes. Collectively, we propose *GRB7* as a novel therapeutic target in ESCC patients having 17q12 amplification. Moreover, since *GRB7* reportedly acts with other tyrosine kinase receptors as well as *ERBB2*, we expect that inhibition of *GRB7* would be a novel therapeutic strategy effective for ESCC patients with resistance to trastuzumab.

Overall, this study provides important insights into the pathogenesis of ESCC; especially we found *GRB7* as a novel ESCC driver gene and potential new therapeutic target. This fruitful result proves usefulness of our integrative analysis approach in screening for therapeutic targets in cancer research.

## Supporting Information

S1 Fig Summary of samples and experiments in the discovery set. The colored table shows different subsets of 83 samples, which were subjected to microarray expression profiling and array-CGH.
(TIF)

S2 Fig The results of GISTIC analysis on SNP array data in GEO (accession number: GSE 17958). The red box indicates significant peaks that were also observed in our aCGH data.
(TIF)

S3 Fig Results of integrative analysis and G scores obtained from GISTIC. The y-axis on the left side represents the G score, while the y-axis on the right side represents the *p*-value of the Wilcoxon test in the edira algorithm. G scores of positive values indicate amplification, while G scores of negative values indicate deletion. The red and blue lines show G scores of amplifications and deletions, respectively. The dotted red line indicates the threshold of amplification, while the dotted blue line indicates the threshold of deletion. Dot plots indicate *p*-values of correlations between expression and copy number alterations calculated by Wilcoxon test in each position. Dot plots in light blue and orange represent correlation directions of amplification and deletion, respectively. The regions in yellow indicate significant correlations between expression and copy number alterations.
(TIF)

S1 File The effects of *ERBB2* knockdown and trastuzumab treatment in ESCC cell lines. (Figure A) Reductions in mRNA and protein levels of ERBB2 at 48 hours after siRNA transfection in KYSE410 and TE4 cells. The results are the mean ± SD from 3 replicates of a single experiment. (Figure B) *ERBB2* inactivation reduced proliferation of KYSE410 and TE4 cells. Cell growth was measured on days 2, 3, and 4 by MTT assay. Absorbance at day 0 was assigned a value of 1. The results are the mean ± SD from 6 replicates of a single experiment. (Figure C) Inhibitory effects of trastuzumab on ESCC cells. Dose-response curves of trastuzumab in KYSE410 and TE4 cells. Cells were treated with the indicated concentrations of trastuzumab for 72 hours. Cell growth inhibition was analyzed by MTT assay, as described in the Materials and Methods. Ratios of the mean absorbance of wells containing drug over the mean absorbance of drug-free wells were plotted against different concentrations of trastuzumab. The results are the means ± SDs from 6 replicates of a single experiment.
(TIF)

S1 Table Individual clinical data for the discovery set. (DOCX)

S2 Table *GRB7* mRNA expression and clinicopathological factors in the discovery set. (DOCX)

S3 Table Individual clinical data for the validation set. (DOCX)

S4 Table Chromosomal arm level events in ESCC. (TIF)

S5 Table *GRB7* mRNA expression and clinicopathological factors in the validation set. (DOCX)
