# Supplementary figures and images for: An Integrative Analysis to Identify Driver Genes in Esophageal Squamous Cell Carcinoma
Source: PLoS One. 2015 Oct 14;10(10):e0139808. doi: 10.1371/journal.pone.0139808 (PMC4605796; doi:10.1371/journal.pone.0139808)

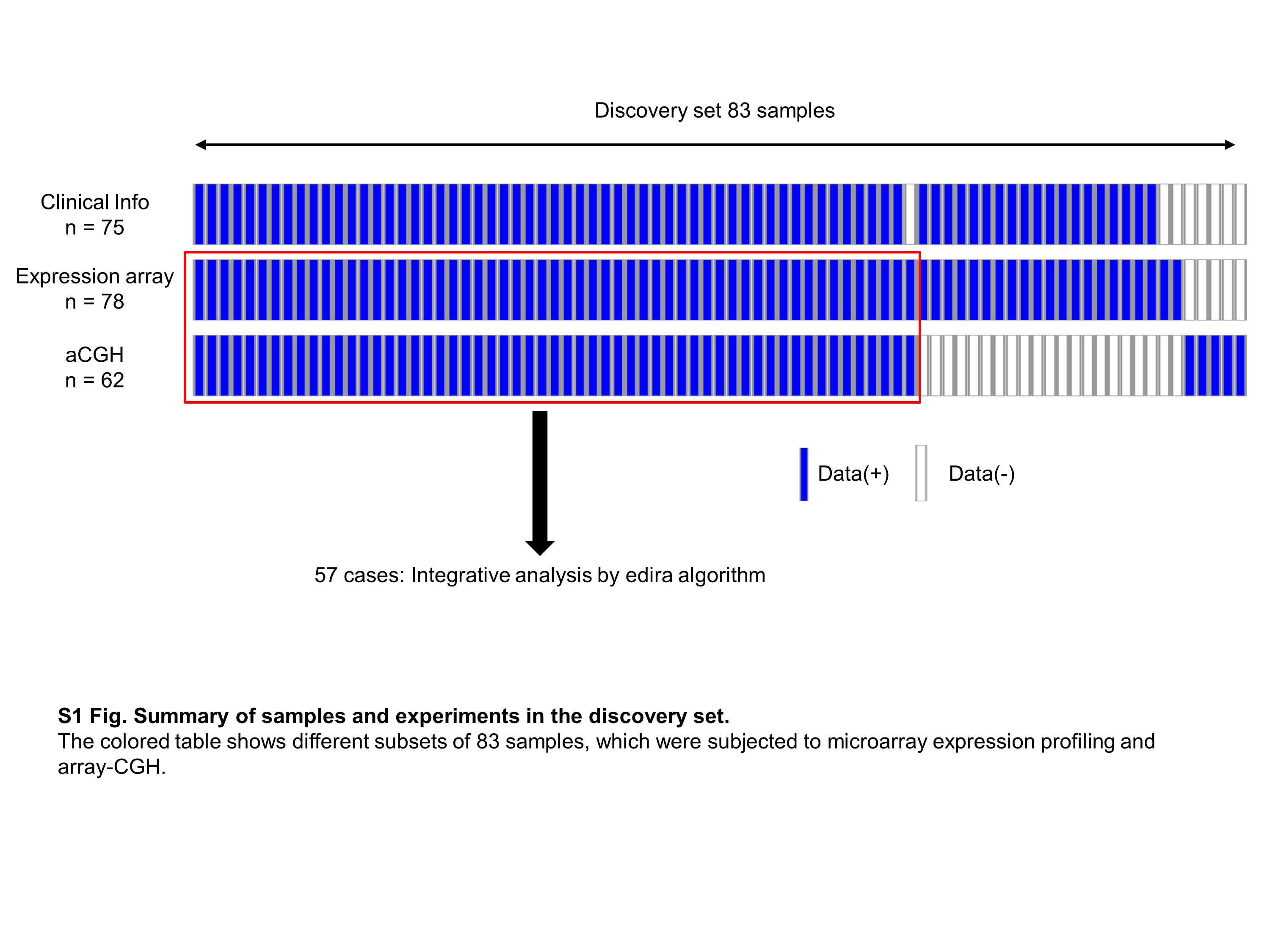

Supplement: S1 Fig — The colored table shows different subsets of 83 samples, which were subjected to microarray expression profiling and array-CGH. (TIF) [file pone.0139808.s001.tif]

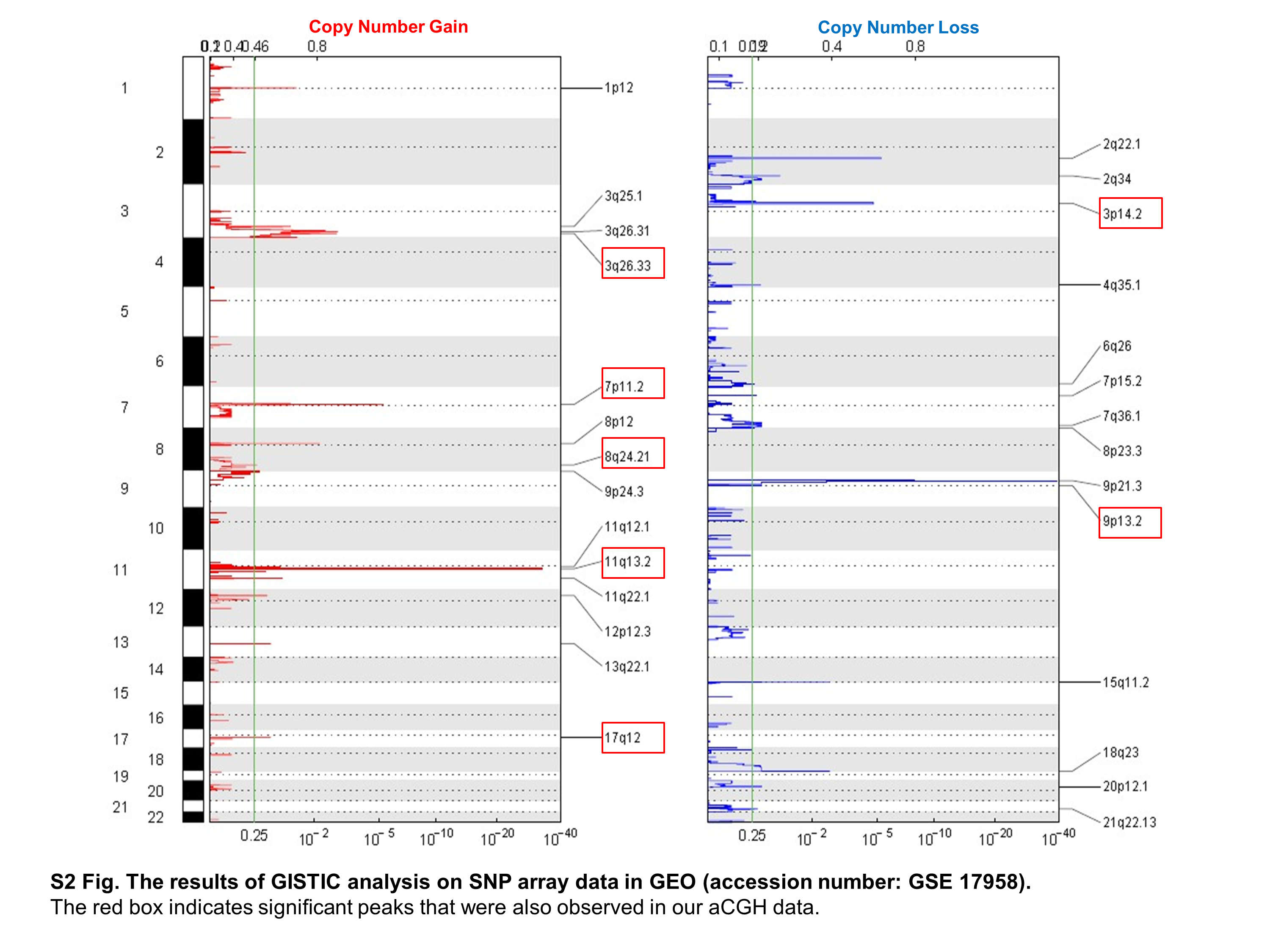

Supplement: S2 Fig — The red box indicates significant peaks that were also observed in our aCGH data. (TIF) [file pone.0139808.s002.tif]

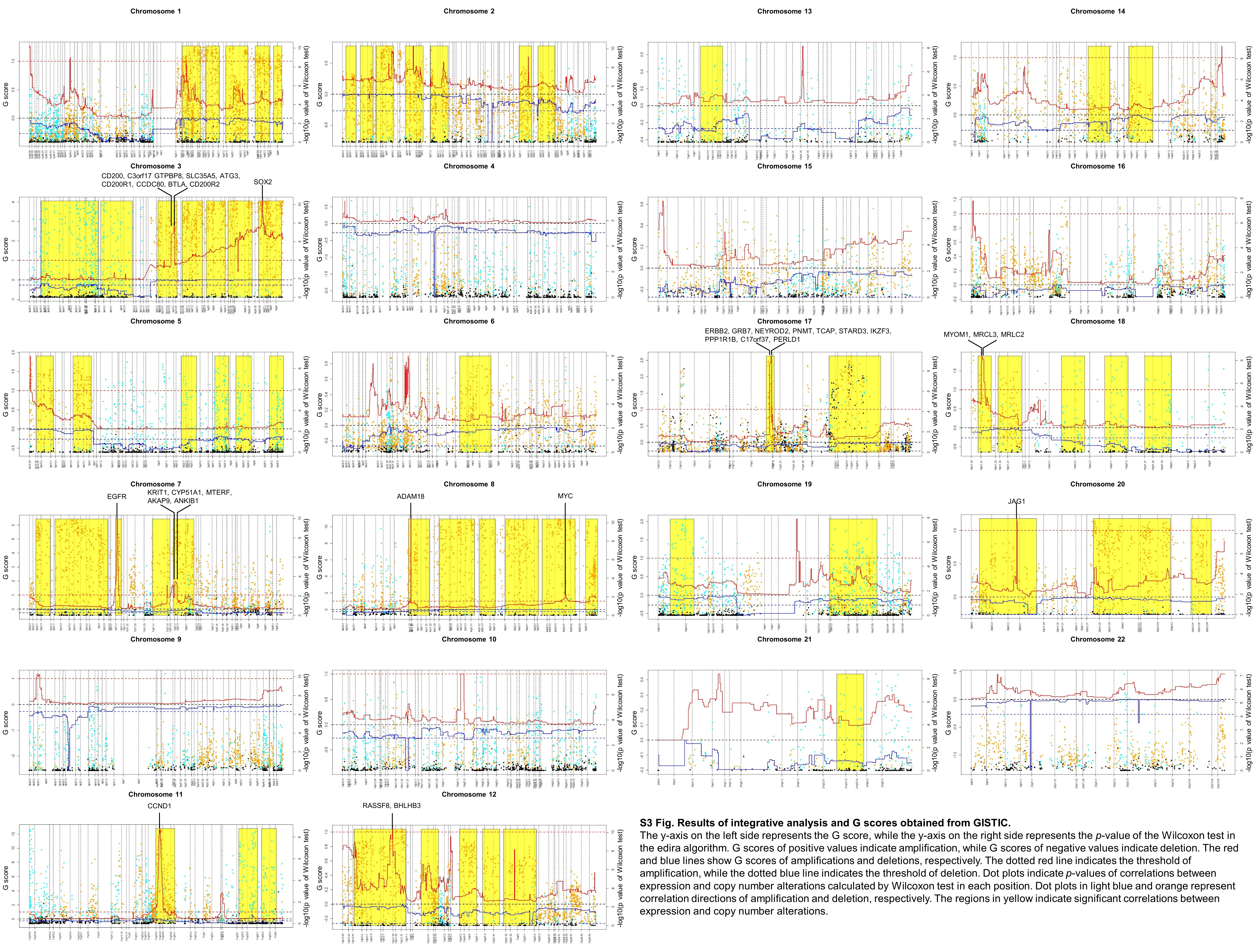

Supplement: S3 Fig — The y-axis on the left side represents the G score, while the y-axis on the right side represents the p-value of the Wilcoxon test in the edira algorithm. G scores of positive values indicate amplification, while G scores of negative values indicate deletion. The red and blue lines show G scores of amplifications and deletions, respectively. The dotted red line indicates the threshold of amplification, while the dotted blue line indicates the threshold of deletion. Dot plots indicate p-values of correlations between expression and copy number alterations calculated by Wilcoxon test in each position. Dot plots in light blue and orange represent correlation directions of amplification and deletion, respectively. The regions in yellow indicate significant correlations between expression and copy number alterations. (TIF) [file pone.0139808.s003.tif]

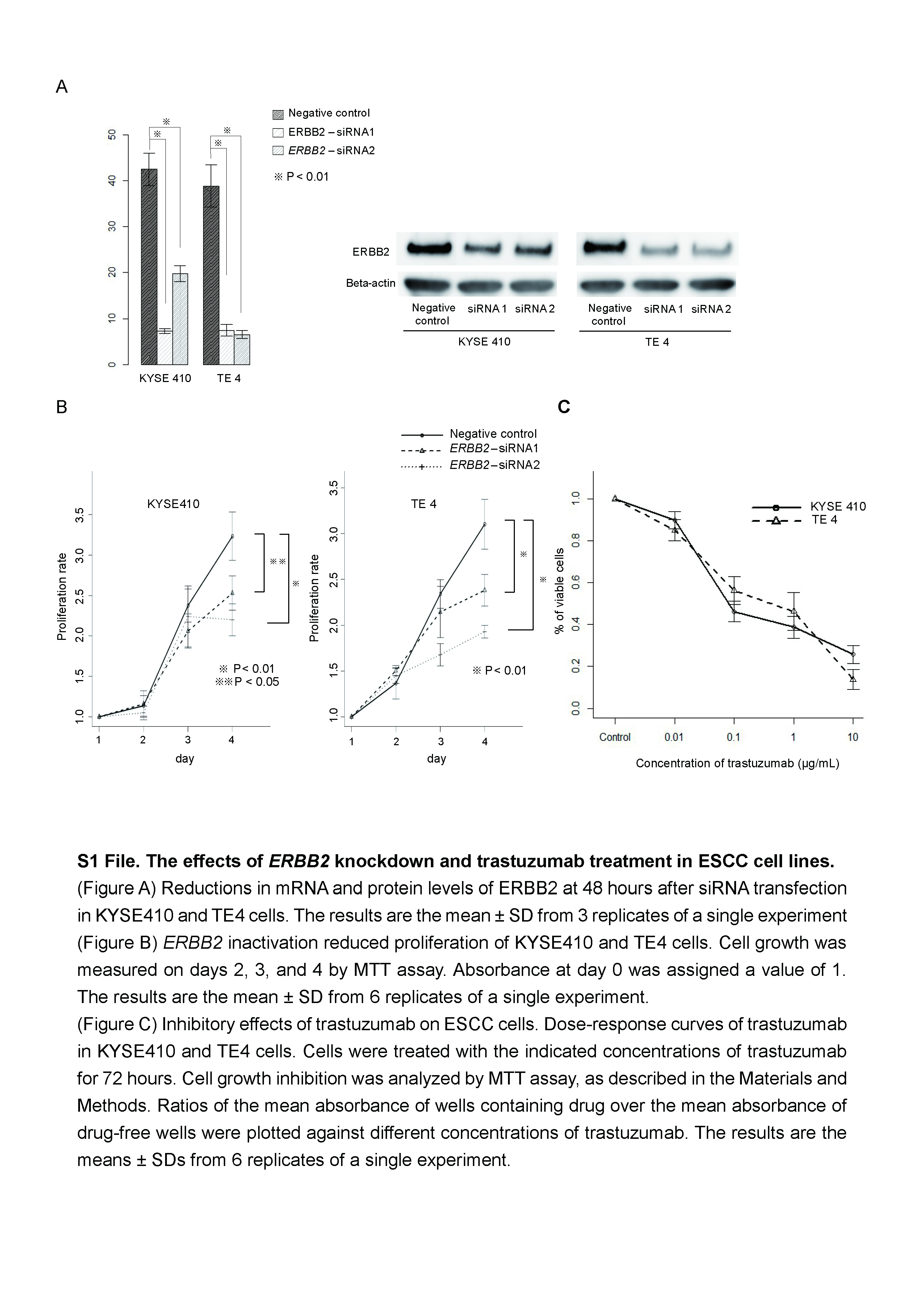

Supplement: S1 File — (Figure A) Reductions in mRNA and protein levels of ERBB2 at 48 hours after siRNA transfection in KYSE410 and TE4 cells. The results are the mean ± SD from 3 replicates of a single experiment. (Figure B) ERBB2 inactivation reduced proliferation of KYSE410 and TE4 cells. Cell growth was measured on days 2, 3, and 4 by MTT assay. Absorbance at day 0 was assigned a value of 1. The results are the mean ± SD from 6 replicates of a single experiment. (Figure C) Inhibitory effects of trastuzumab on ESCC cells. Dose-response curves of trastuzumab in KYSE410 and TE4 cells. Cells were treated with the indicated concentrations of trastuzumab for 72 hours. Cell growth inhibition was analyzed by MTT assay, as described in the Materials and Methods. Ratios of the mean absorbance of wells containing drug over the mean absorbance of drug-free wells were plotted against different concentrations of trastuzumab. The results are the means ± SDs from 6 replicates of a single experiment. (TIF) [file pone.0139808.s004.tif]

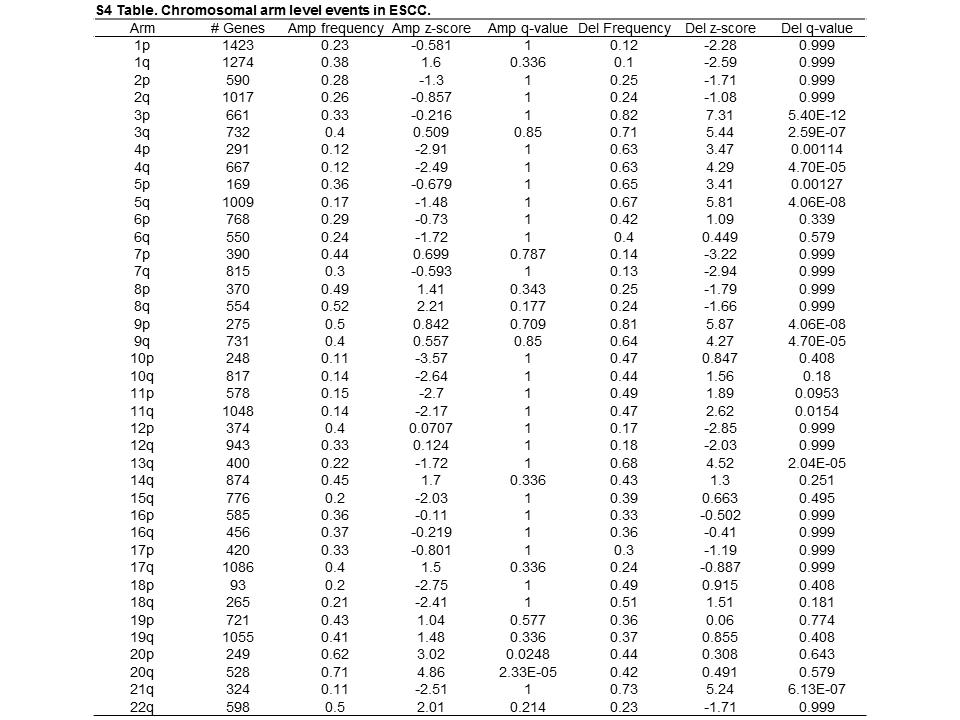

Supplement: S4 Table — (TIF) [file pone.0139808.s008.tif]
