# Supplementary material for: An Integrative Analysis to Identify Driver Genes in Esophageal Squamous Cell Carcinoma
Source: PLoS One. 2015 Oct 14;10(10):e0139808. doi: 10.1371/journal.pone.0139808 (PMC4605796; doi:10.1371/journal.pone.0139808)
Supplement: S1 Table — (DOCX) [file pone.0139808.s005.docx]

**S1 Table. Individual clinical data for the discovery set*.**

well: well differentiated squamous cell carcinoma, mod: moderately differentiated squamous cell carcinoma, poor: poorly differentiated squamous cell carcinoma

* Information on clinicopathological factors were available for 75 out of 83 patients in the discovery set (the detail is shown in Figure S1).
