# Supplementary material for: An Integrative Analysis to Identify Driver Genes in Esophageal Squamous Cell Carcinoma
Source: PLoS One. 2015 Oct 14;10(10):e0139808. doi: 10.1371/journal.pone.0139808 (PMC4605796; doi:10.1371/journal.pone.0139808)
Supplement: S2 Table — (DOCX) [file pone.0139808.s006.docx]

**S2 Table. *GRB7* mRNA expression and clinicopathological factors in the discovery set^*^.**

| **Factors** |  | **High expression** | **Low expression** | **P value** |
| --- | --- | --- | --- | --- |
|  |  | **n = 14** | **n = 61** |  |
| Age (mean ± SD) |  | 64.8±9.3 | 66.56±7.99 | 0.53 |
| Sex | Male:Female | 13 :1 | 53 : 8 | 0.66 |
| Histology | well | 7 | 19 | 0.21 |
|  | mod＆poor | 7 | 42 |  |
| Depth | T1 | 3 | 7 | 0.38 |
|  | T2-T4 | 11 | 54 |  |
| Lymph node metastasis | Negative | 4 | 26 | 0.38 |
|  | Positive | 10 | 35 |  |
| Lymphatic invasion | Negative | 5 | 20 | 1 |
|  | Positive | 9 | 41 |  |
| Venous invasion | Negative | 3 | 14 | 1 |
|  | Positive | 11 | 47 |  |

SD: standard deviation, well: well differentiated squamous cell carcinoma, mod: moderately differentiated squamous cell carcinoma, poor: poorly differentiated squamous cell carcinoma.

* Information on clinicopathological factors were available for 75 out of 83 patients in the discovery set (the detail is shown in Figure S1).
