# Supplementary material for: An Integrative Analysis to Identify Driver Genes in Esophageal Squamous Cell Carcinoma
Source: PLoS One. 2015 Oct 14;10(10):e0139808. doi: 10.1371/journal.pone.0139808 (PMC4605796; doi:10.1371/journal.pone.0139808)
Supplement: S3 Table — (DOCX) [file pone.0139808.s007.docx]

**S3 Table. Individual clinical data for the validation set.**

well: well differentiated squamous cell carcinoma, mod: moderately differentiated squamous cell carcinoma, poor: poorly differentiated squamous cell carcinoma
