# Supplementary material for: An Integrative Analysis to Identify Driver Genes in Esophageal Squamous Cell Carcinoma
Source: PLoS One. 2015 Oct 14;10(10):e0139808. doi: 10.1371/journal.pone.0139808 (PMC4605796; doi:10.1371/journal.pone.0139808)
Supplement: S5 Table — (DOCX) [file pone.0139808.s009.docx]

**S5 Table. *GRB7* mRNA expression and clinicopathological factors in the validation set.**

| **Factors** |  | **High expression** | **Low expression** | **P value** |
| --- | --- | --- | --- | --- |
|  |  | **n = 25** | **n = 60** |  |
| Age (mean ± SD) |  | 65.9±7.2 | 64.5±8.5 | 0.45 |
| Sex | Male:Female | 21 : 4 | 53 : 7 | 0.72 |
| Histology | well | 7 | 23 | 0.45 |
|  | mod & poor | 18 | 37 |  |
| Depth | T1 | 4 | 5 | 0.43 |
|  | T2-T4 | 21 | 55 |  |
| Lymph node metastasis | Negative | 3 | 20 | 0.06 |
|  | Positive | 22 | 40 |  |
| Lymphatic invasion | Negative | 2 | 11 | 0.32 |
|  | Positive | 23 | 49 |  |
| Venous invasion | Negative | 3 | 7 | 1 |
|  | Positive | 22 | 53 |  |

SD: standard deviation, well: well differentiated squamous cell carcinoma, mod: moderately differentiated squamous cell carcinoma, poor: poorly differentiated squamous cell carcinoma.
